# miR-155-overexpressing monocytes resemble HLA^high^ISG15^+^ synovial tissue macrophages from patients with rheumatoid arthritis and induce polyfunctional CD4^+^ T-cell activation

**DOI:** 10.1093/cei/uxab016

**Published:** 2021-11-30

**Authors:** Anton M Olsson, Giovanni A M Povoleri, Domenico Somma, Michael L Ridley, Tatiana Rizou, Sylvine Lalnunhlimi, Lucy Macdonald, Megha Rajasekhar, Rocio T Martinez-Nunez, Mariola Kurowska-Stolarska, Leonie S Taams

**Affiliations:** 1 Centre for Inflammation Biology and Cancer Immunology, Department of Inflammation Biology, School of Immunology and Microbial Sciences, King’s College London, London, UK; 2 Institute of Infection, Immunity, and Inflammation, University of Glasgow, Glasgow, UK; 3 Research into Inflammatory Arthritis Centre Versus Arthritis (RACE), University of Glasgow, Glasgow, UK; 4 Department of Infectious Diseases, School of Immunology & Microbial Sciences, King’s College London, London, UK; 5 GSK, Stevenage, UK; 6 Francis Crick Institute, London, UK; 7 Melbourne School of Population and Global Health, The University of Melbourne, Carlton, VIC, Australia

**Keywords:** microRNA, innate immunity, monocyte, immune regulation, IL-10

## Abstract

MicroRNAs (miRs) are known to regulate pro-inflammatory effector functions of myeloid cells, and miR dysregulation is implicated in rheumatoid arthritis (RA), a condition characterized by inflammation and destruction of the joints. We showed previously that miR-155 is increased in myeloid cells in RA and induces pro-inflammatory activation of monocytes and macrophages; however, its role at the interface between innate and adaptive immunity was not defined. Here, RNA-sequencing revealed that overexpression of miR-155 in healthy donor monocytes conferred a specific gene profile which bears similarities to that of RA synovial fluid-derived CD14^+^ cells and HLA^high^ISG15^+^ synovial tissue macrophages, both of which are characterized by antigen-presenting pathways. In line with this, monocytes in which miR-155 was overexpressed, displayed increased expression of HLA-DR and both co-stimulatory and co-inhibitory molecules, and induced activation of polyfunctional T cells. Together, these data underpin the notion that miR-155-driven myeloid cell activation in the synovium contributes not only to inflammation but may also influence the adaptive immune response.

## Teaser

miR-155 overexpression of healthy control blood monocytes confers a specific gene signature that bears similarities to that of RA synovial macrophages. miR-155-overexpressing monocytes induce polyfunctional CD4^+^T-cell activation. This study reaffirms and extends the association of miR-155 and joint inflammation in RA.

## Graphical Abstract

**Graphical Abstract:**
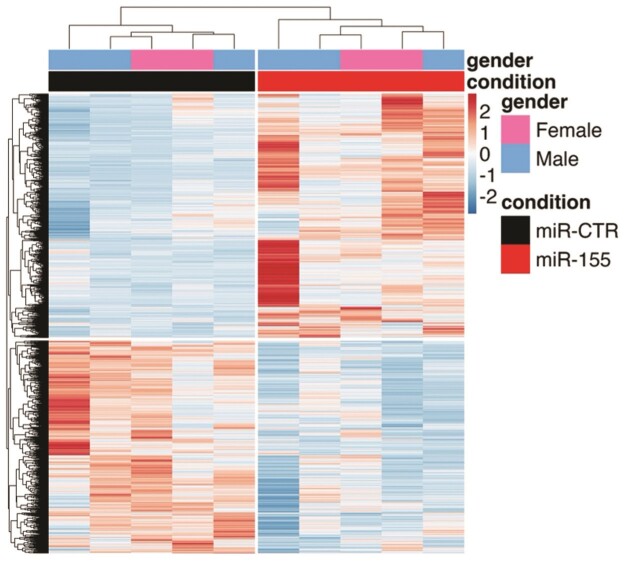

## Introduction

Rheumatoid arthritis (RA) is a chronic autoimmune disease characterized by inflammation of the synovium and subsequent destruction of cartilage and bone of the joints. A broad range of cells and mediators of both the innate and adaptive immune systems contribute to RA pathogenesis. This knowledge has led to the identification of several immune-based therapies including inhibition of cytokine signalling such as TNFα, IL-6 and JAK/STAT pathways, B-cell depletion, and the blockade of co-stimulatory molecules. Monocytes and macrophages are innate immune cells that have a plethora of functions including antigen presentation and orchestration of the adaptive immune response. They play a crucial role in RA pathogenesis: these cells can accumulate in the synovial tissue (ST), produce pro-inflammatory cytokines, differentiate into osteoclasts and promote Th1 and Th17 cell polarization [1–7]. Recent research has shown that different subsets of monocytes and macrophages with distinct roles are present in the mouse and human joint and can contribute differently to inflammation [8–13]. It was shown in mice that CX3CR1^+^ tissue-resident macrophages form a protective immunological barrier at the synovial lining [10]. This barrier is disrupted in experimental arthritis when circulating blood monocyte populations are recruited that can drive and maintain inflammatory arthritis [8, 9]. More recently, single-cell RNA-sequencing analysis of ST from healthy donors and patients with RA revealed distinct subsets of synovial tissue macrophages (STM) some of which were associated with treatment-resistant RA or with disease remission [11]. Understanding how the fate and state of monocytes and macrophages are regulated in RA is therefore of biological and clinical relevance.

MicroRNAs (miRNAs) are small non-coding RNA molecules (~22 nt in length) that modulate gene expression post-transcriptionally. miRNAs are involved in the regulation of a wide range of cellular and biological processes, including proliferation, differentiation, and survival. A single miRNA can target multiple different mRNA molecules and negatively modulate their expression, thereby altering downstream networks and pathways, eventually resulting in a significant change in cell phenotype [14]. Dysregulation of miRNA expression has been described in various disease states including inflammation, cardiovascular disease, cancer, and autoimmunity [15]. MicroRNA-155-5p (miR-155 herein) is one of the most studied miRNAs; it plays an important role in the function and development of immune cell subsets including myeloid cells and its dysregulation has been described in autoimmune diseases, including RA [7]. A critical observation was that miR-155 KO mice do not develop collagen-induced arthritis [16]. In addition, peripheral blood monocytes (PBM) from patients with RA contain increased copy numbers of miR-155 which correlate with disease activity [17]. Increased expression levels of miR-155 were also detected in CD14^+^ cells from the synovial fluid of patients with RA as compared to their peripheral blood counterparts [16–18]. Several target genes have been validated for miR-155 including *INPP5D* encoding SHIP-1, a potent inhibitor of inflammatory pathways [16, 19], suppressor of cytokine signalling 1 (*SOCS1*), a negative regulator of type I IFN signalling [20], and *BCL6* which inhibits pro-inflammatory NF-κB signalling [21], all leading to an increased pro-inflammatory response. In line with these findings, overexpression of miR-155 in CD14^+^ monocytes was shown to result in the production of pro-inflammatory cytokines (TNFα, IL-6, IL-8, and IL-1β) [16], chemokines (CCL3, CCL4, CCL5, and CCL8) [17], and increased resistance to spontaneous apoptosis [18]. Together these studies provide strong evidence that upregulated miR-155 expression in monocytes contributes to inflammation in RA. To further explore the role of miR-155 in RA, we investigated the transcriptional and phenotypic consequences of *in vitro* miR-155 overexpression in monocytes, and how these relate to the *in vivo* gene signatures of RA synovial macrophage subsets. We also examined how miR-155-overexpressing monocytes affect CD4^+^ T-cell activation or polarization. Our data show that miR-155-overexpressing monocytes have a gene signature similar to that of synovial macrophage subsets present in treatment-resistant RA, and promote polyfunctional CD4^+^ T-cell activation.

## Materials and methods

### Patients and samples

Peripheral blood (PB) was obtained from healthy adult volunteers at King’s College London. Healthy volunteers were between the ages of 18–60 years (mean age ± SD: 28 ± 8 years, male/female 31/35). PB and synovial fluid (SF) samples were obtained from patients with RA or psoriatic arthritis (PsA) attending the Guy’s and St Thomas’ NHS Foundation Trust Rheumatology outpatient clinics. Patient demographic and clinical data are summarized in Supplementary Table 1, and a flow chart indicating in which assays the patient samples were analysed is shown in Supplementary Figure 1. All donors provided written informed consent. This study was approved by the Bromley Ethics Research Committee (06/Q0705/20).

### Cell isolation

Peripheral blood mononuclear cells (PBMC) and synovial fluid mononuclear cells (SFMC) were isolated using density gradient centrifugation with Lymphoprep™ (Axis-Shield, Oslo, Norway). Healthy volunteer PBMC were isolated by MACS isolation using anti-CD14 positive selection kits (Miltenyi Biotec, Bergisch-Gladbach, Germany) (purity > 90%). Patient-derived CD14^+^ monocytes were isolated from PBMC and SFMC by MACS isolation (as above); on some occasions, CD14^+^ monocytes were isolated from paired PBMC and SFMC by FACS sorting on a BDAriaII following staining with an anti-CD14-APC-Cy7 antibody (Clone HCD14; Biolegend, San Diego, CA, USA) (purity > 98%). CD4^+^ T cells were negatively selected from the CD14^−^ flowthrough using MACS CD4^+^ T-cell isolation kits (Miltenyi Biotec, Bergisch-Gladbach, Germany) (purity > 95%).

### Transfection

Transfection of CD14^+^ monocytes was performed using the NTER nanoparticle transfection reagent (Sigma-Aldrich, St. Louis, MO, USA) following manufacturer’s instructions. Monocytes were plated at 0.5 × 10^6^/well, 48-well plate, at a final volume of 250 µl and transfected with a final concentration of 20 nM of miR-155 MiRIDIAN miRNA mimic (mature sequence: UUAAUGCUAAUCGUGAUAGGGGUU, Horizon Dharmacon) or negative control mimic (Horizon Dharmacon) or mirVana negative control (sequence: proprietary, ThermoFisher) for 4 or 24 h. To measure transfection efficiency, separate cells were transfected with a Dy547 fluorophore-conjugated miR-155 mimic or negative control mimic (20 nM, Horizon Dharmacon), which was read out on the FITC channel.

### Monocyte stimulation assays

To assess miR-155 expression upon monocyte stimulation, MACS-isolated CD14^+^ monocytes were plated in a 48-well plate (1 × 10^6^/well) in a final volume of 1 ml of complete medium [RPMI 1640 (Life Technologies, Carlsbad, CA, USA), 10% FCS (Gibco), 1% Pen/Strep (Life Technologies), and 2 mM l-glutamine (Life Technologies)]. Monocytes were stimulated with LPS (100 ng/ml, Sigma-Aldrich) or with IL-1β (10 ng/ml, Peprotech, UK) + IL-6 (10 ng/ml, Peprotech) + TNFα (10 ng/ml, Miltenyi), and incubated at 37°C, 5% CO_2_ for 24 h. For some experiments, monocytes were cultured in the presence of cell-free SF from patients with active RA (obtained by spinning down 1 ml SF for 3.5 min at 8000 rpm, and storing the supernatant at −80°C) at 0, 1, 5, or 10% v/v and incubated at 37°C, 5% CO_2_ for 24 h. Following culture, cells were put on ice for 30 min before removal from the plate for RNA extraction. Cell-free culture supernatants were stored at −80°C for ELISA.

### Monocyte phenotype assays

To assess how miR-155 affected monocyte phenotype, CD14^+^ monocytes were plated in a 48-well plate (0.5 × 10^6^/well) in a final volume of 250 μl of complete medium and transfected with miR-155 or negative control mimic for 24 h. Following transfection, cells were placed on ice for 30 min and collected, washed in FACS buffer (PBS, 0.5% BSA, 0.1% NaN_3_) and stained with CD14-APC-Cy7 (clone TUK4, Miltenyi), HLA-DR-PerCP-Cy5.5 (clone G46-6, BD Bioscience), CD86-PacBlue (clone IT2.2, Biolegend), CD80-FITC (clone 2D10, Biolegend), PD-L1-PE (clone H1M1, BD Bioscience), and PD-L2-APC (clone MIH18, BD Bioscience) for 30 min. Cells were washed with FACS buffer [PBS + 1% BSA (Sigma)] and fixed in 2% paraformaldehyde (Merck Millipore) for 15 min, then washed once, resuspended in FACS buffer and acquired on a BD FACS Canto II.

### Monocyte/CD4^+^ T-cell co-culture assays

For co-culture experiments, CD14^+^ monocytes were plated in a 48-well plate (0.5 × 10^6^/well) in a final volume of 250 μl of complete medium and transfected with miR-155 or negative control mimic for 4 h. Following transfection, monocytes were washed in complete media and re-plated (0.5 × 10^6^ cells/well, 48-well plate, total volume 1 ml) together with autologous CD4^+^ T cells at a 1:1 ratio in the presence of 100 ng/ml anti-CD3 mAb (OKT3) (BioLegend, UK). Cells were cultured for 72 h. After culture, cells were restimulated with PMA (50 ng/ml), ionomycin (750 ng/ml), and Golgistop for 3 h prior to staining. Cells were stained extracellularly with CD14-APC-Cy7 and then washed with 0.5% saponin (Sigma-Aldrich) followed by intracellular staining with anti-CD3-PE-Cy7 (UCHT1, Biolegend), CD4-PerCP-Cy5.5 (clone SK3, Biolegend), TNFα-BV605 (clone Mab11, Biolegend), IFNγ-PacBlue (clone 4S.B3, Biolegend), IL-4-AF647 (clone 8D4-8, Biolegend), IL-10-AF488 (clone JES3-9D7, Biolegend), and IL-17A-PE (clone BL168, Biolegend) for 30 min. Following staining, cells were washed twice in 0.5% saponin and resuspended in FACS buffer and acquired on BD FACS Canto II or Fortessa. Flow cytometry data were analysed using Flow Jo version 10.7.1 (FlowJo, USA). In some experiments, monocyte/CD4^+^ T-cell co-culture experiments were performed in the presence of 1 µg/ml of the PD1-blocking mAb pembrolizumab (Merck) or isotype control (Abcam, UK). CD4^+^ T cells were then stained extracellularly with PD1-APC (clone PD1.3.1.3, Miltenyi) prior to intracellular staining for IL-10 and IFNγ.

### SPICE analysis

Flow cytometry data were analysed by Boolean combination gating on single IFNγ, TNFα, IL-17A, IL-10, and IL-4 positive cells in FlowJo v10.7.1, resulting in 31 different cytokine combination profiles. Frequencies for all combinations were imported into SPICE [22] software for cytokine co-expression analysis and visualization.

### qPCR

Total RNA was extracted using QIAzol reagent (Qiagen, Hilden, Germany) and the miRNeasy micro kit (Qiagen). Mature miRNA levels were quantified on an ABI7900HT machine using MicroRNA RT kit and TaqMan miRNA assays (Life Technologies) for miR-155-5p and the housekeeping RNAs RNU44 and RNU48, following manufacturer’s protocols.

### ELISA

Cell-free culture supernatants were assessed for the presence of the cytokines IL-6, IL-8, TNFα, and IL-10 using ELISA MAX kits (Biolegend) according to the manufacturer’s protocols. Plates were read at 450 nm using a Spark 10M (Tecan, Männedorf, Switzerland).

### RNA-sequencing of miR-155-transfected CD14^+^ monocytes

MACS-isolated CD14^+^ monocytes from healthy controls were transfected with miR-155 or negative control mimic for 24 h. Following transfection, cells were stained with CD14-APC-Cy7 and live-dead dye, followed by FACS sorting on a BD FACS Aria of live CD14^high^ FSC^hi^ cells (which are enriched for transfected cells; Supplementary Figure 2), and RNA extraction. Total RNA (RNA integrity number > 7) was used to construct RNA-sequencing libraries. Starting with 100 ng total RNA, cDNA synthesis and amplification were performed using a NEBNext Ultra II Directional RNA Library Prep Kit for Illumina (NEB, Ipswich, MA, USA) following the manufacturer’s protocol. Dual-indexed libraries were prepared using the NEBNext® Multiplex Oligos for Illumina® (Dual Index Primers Set 1) following the manufacturer’s protocol. The libraries were sequenced at Genewiz Ltd (Genewiz, Germany) on an Illumina NovaSeq 2 × 150 bp at a median 46.8 M reads per sample. The reads were mapped against genome using STAR on default parameters against the human hg38 reference genome release 32. Differential gene expression analysis (adjusted *P* < 0.05 and a log fold change > ±2) was performed using DESeq2. Raw data can be accessed at GEO [GSE166605].

### Microarray analysis of PB and SF CD14^+^ from patients with RA

Publicly available data sets of the gene expression profiles of CD14^+^ cells from RA PB vs. SF (E-MEXP-3890 [23] and GSE71370 [18]) were downloaded and analysed for differential expression using Partek® Genomics Suite®. A gene list was generated representing all genes differentially up- or downregulated (2-fold threshold) in SF vs. PB RA CD14^+^ cells with an adjusted *P* < 0.05, containing 2070 upregulated and 1625 downregulated genes in RA SF vs. PB CD14^+^ cells.

### Gene Set Enrichment Analysis and scRNA-seq comparison

Gene Set Enrichment Analysis (GSEA) was performed using GSEA version 4.1.0. from the Broad Institute [24] on DESeq2 normalized counts from the RNA-seq data sets of the miR-155-overexpressing monocytes; gene sets for synovial fluid-derived CD14^+^ cells were derived from E-MEXP-3890 [23] and GSE71370 [18]; gene sets for STM subpopulations were sourced from Alivernini et al. [11]. Inclusion gene set size was set between 1 and 5000 and the phenotype was permutated 10 000 times. Gene sets that met the false discovery rate < 0.05 were considered.

CIBERSORTx [25] was used to deconvolute bulk RNA-seq data and provide an estimate of the cell composition, querying the STM subpopulations [11]. scRNA-seq data were downsampled to 300 cells per cluster, signature matrix was created using quartile normalization, min expression 0.1 and cell fractions imputation was performed with batch correction S-mode and 1000 permutation.

To mitigate the dropout problem for miR-155 violin plots expression, imputation was performed using ALRA algorithm [26] included in the Seurat package.

### Statistical analysis

GraphPad Prism (8.1.1, GraphPad, San Diego, CA, USA) was used for statistical analyses. R was employed to perform DESeq2 and Seurat (CRAN repositories). Samples with *n* < 8 were tested non-parametrically and samples *n* > 8 were tested for normality using D’Agostino and Pearson omnibus normality test and then tested for significance using the appropriate parametric or non-parametric test as stated in the figure legends. For SPICE analysis, statistical significance was calculated using a Wilcoxon signed-rank test.

## Results

### Overexpression of miR-155 confers a specific gene signature in CD14^+^ monocytes that overlaps with synovial fluid macrophages and HLA^high^ISG15^+^ STM in RA

MiR-155 expression was increased in RA synovial fluid-derived CD14^+^ cells compared to their PBM counterparts, in line with previous reports [16–18] (Supplementary Figure 3A). Our data show that this increased synovial expression was also observed in another rheumatologic disease, PsA (Supplementary Figure 3B). We confirmed that miR-155 expression could be positively modulated in PB monocytes from healthy volunteers by LPS, or by a combination of TNF/IL-6/IL-1β cytokines, which are known to be upregulated in the RA and PsA joint (Supplementary Figure 3C). We also found that miR-155 expression was increased in a dose-dependent manner when healthy control blood monocytes were cultured in the presence of cell-free synovial fluid, particularly those from patients not receiving any drug treatment (Supplementary Figure 3D). Production of IL-6 was measured to confirm that synovial fluid treatment induced an inflammatory response in CD14^+^ monocytes; similar to miR-155 expression there was more IL-6 production when monocytes were cultured in the presence of synovial fluid from patients not receiving any treatment (Supplementary Figure 3E).

**Fig. 1.**
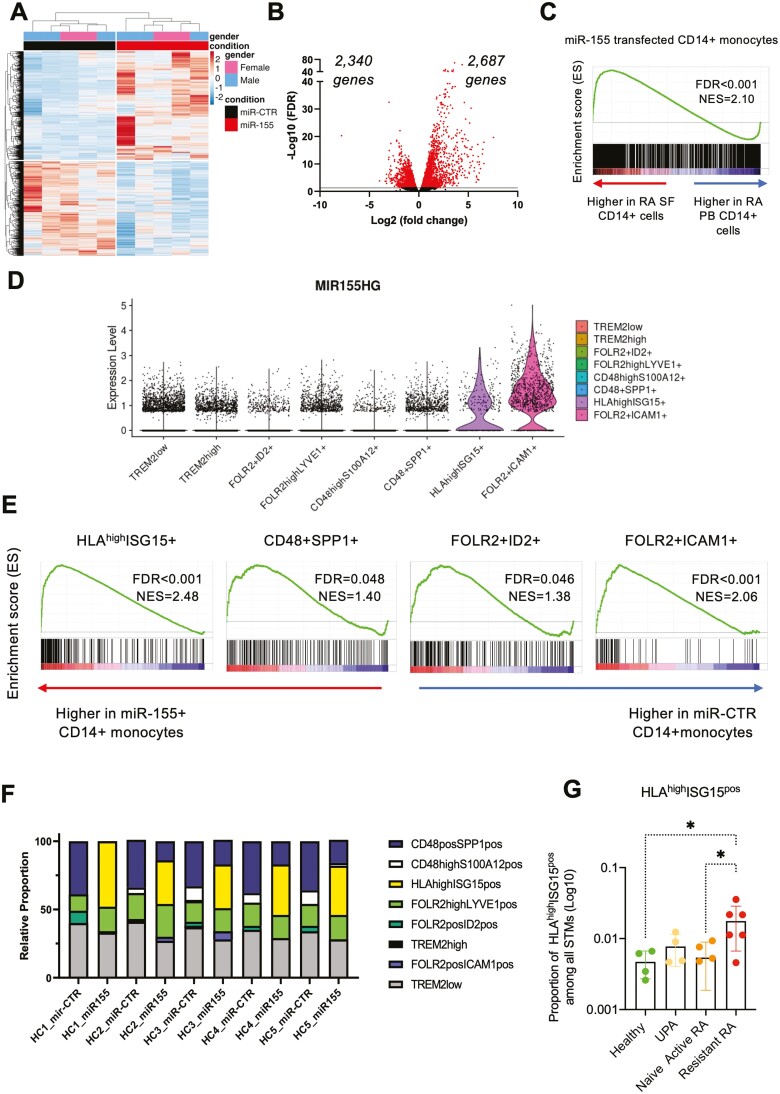
miR-155 overexpression in CD14^+^ monocytes confers a specific gene signature that overlaps with synovial macrophages in active RA. CD14^+^ monocytes were transfected for 24 h with miR-155 or negative control mimic (miR-CTR), after which RNA was isolated for RNA-seq. (A) Heatmap of all differentially expressed genes in miR-155-transfected CD14^+^ monocytes compared to negative control mimic. (B) Volcano plot of differentially expressed genes. Grey line represents a false discovery rate (FDR) of 0.05 and red data points represent genes that have a FDR of <0.05. (C) GSEA plots of genes associated with miR-155-transfected CD14^+^ monocytes in relation to the gene expression profile of RA SF (vs. PB) CD14^+^ monocytes. (D) Violin plots showing the imputed expression of MIR155HG in different synovial macrophage subsets. (E) GSEA plots of genes associated with the RA STM subsets HLA^high^ISG15^+^, CD48^+^SPP1^+^, FOLR2^+^ID2^+^, and FOLR2^+^ICAM1^+^ in relation to the gene expression profile of miR-155 (vs. negative control miR mimic)-transfected CD14^+^ monocytes. Graphs show multiple-test adjusted FDR-corrected *q*-values and normalized expression score (NES). (F) Relative proportion of different synovial macrophage subpopulations (as based on the associated gene signature) deconvoluted from bulk RNA-seq data, after miR-155 mimic or negative control mimic transfection. (G) Proportion of HLA^high^ISG15^+^ synovial macrophage subset present in healthy, early arthritis (UPA), treatment-naïve RA and treatment-resistant RA.

To determine how miR-155 affects the phenotype and function of CD14^+^ monocytes, we transfected healthy control PBM with either miR-155 or negative control mimic (miR-CTR). Transfection with miR-155 resulted on average in 33% transfected cells and led to significant upregulation of mature miR-155 transcript levels (Supplementary Figure 4) and increased cell survival (not shown), as we previously reported [18]. Following transfection, we sorted live monocytes based on live/dead dye staining and FSC^hi^ CD14^high^ expression thereby enriching for transfected cells (Supplementary Figure 2). RNA was extracted and bulk RNA-seq (*n* = 5 per condition) was performed to explore the transcriptional changes induced by miR-155 in myeloid cells. RNA-seq analysis (adjusted *P <* 0.05) revealed that miR-155 overexpression conferred a unique gene signature in CD14^+^ monocytes, with 2687 upregulated and 2340 downregulated genes (Fig. 1A and B). We compared the list of genes downregulated at least 1.2-fold to a list of 147 validated targets of miR-155 [27, 28]. We chose a low 1.2-fold change in gene expression as the effects of miRNA action can be subtle, i.e. small changes in multiple target genes in the same pathway can have a broad global effect. This analysis showed that 25 genes that were significantly downregulated upon miR-155 overexpression were validated miR-155 targets (Supplementary Figure 5A and B). The well-characterized miR-155 target gene *INPP5D* (encoding SHIP-1) was downregulated in all five donors although this did not reach statistical significance (Supplementary Figure 5C).

We next sought to compare the transcriptional profile of miR-155-transfected monocytes with the profiles of monocytes/macrophages present in the RA joint. First, we performed GSEA using publicly available transcriptomic data on CD14^+^ cells from RA synovial fluid [18, 23], which revealed a significant enrichment (*q*-value < 0.001) of the *in vitro* miR-155-associated gene signature within the expression profile of RA SF CD14^+^ cells compared to RA PB CD14^+^ cells (Fig. 1C). These data suggest that miR-155-overexpressing monocytes bear similarities to synovial fluid CD14^+^ cells as the latter are enriched for a miR-155-regulated gene signature. Recently, Alivernini et al. reported the presence of several distinct STM subsets in joints of healthy donors or patients with treatment-naïve, treatment-resistant, or remission RA [11]. To understand the potential role of miR-155 in the biology of these subsets, we examined the expression of miR-155 in STM clusters. We found that two STM clusters expressed miR-155: HLA^high^ISG15^+^ and FOLR2^+^ICAM1^+^ clusters (Fig. 1D). We then investigated whether the gene signatures of distinct STM clusters were particularly enriched within the transcriptomic profile of miR-155-overexpressing monocytes (miR-155-driven pathways). GSEA revealed that four of the STM subset-associated gene signatures were significantly enriched within miR-155-transfected CD14^+^ monocytes. These included HLA^high^ISG15^+^ and FOLR2^+^ICAM1^+^ that showed high levels of miR-155, and two additional clusters: CD48^+^SPP1^+^ and FOLR2^+^ID2^+^ (Fig. 1E). Of these populations, HLA^high^ISG15^+^ and CD48^+^SPP1^+^ are part of the pro-inflammatory MerTK^neg^CD206^neg^ population, and FOLR2^+^ID2^+^ and FOLR2^+^ICAM1^+^ are part of the resolution-associated MerTK^pos^CD206^pos^ population. To understand better the magnitude of the miR-155 signature in these STM clusters, we interrogated the proportional representation of each of these STM populations (as based on their associated gene signatures) within miR-155-overexpressing monocytes. This analysis showed that miR-155-overexpressing monocytes are particularly enriched for genes associated with the pro-inflammatory HLA^high^ISG15^+^ cluster, whilst they display a reduced proportion of genes associated with the CD48^+^SPP1^+^ subset, as compared to negative control mimic-transfected monocytes (Fig. 1F). Interestingly, the frequency of the HLA^high^ISG15^+^ cluster is significantly increased in ST from RA patients with active, treatment-resistant disease as compared to ST from healthy or treatment-naive active RA (Fig. 1G). Together, these data indicate that overexpression of miR-155 in healthy donor monocytes confers a gene profile that bears similarity to that of synovial fluid CD14^+^ cells and HLA^high^ISG15^+^ STM from patients with treatment-resistant, active RA.

### miR-155 overexpression promotes an activated phenotype in CD14^+^ monocytes

**Fig. 2.**
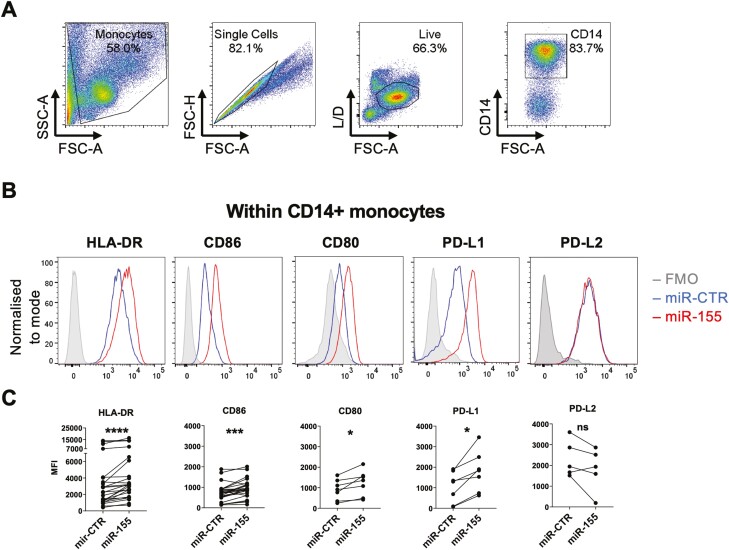
Effect of miR-155 overexpression on CD14^+^ monocyte cell surface phenotype. Healthy donor CD14^+^ monocytes were transfected with negative control or miR-155 mimic and cultured for 24 h. (A) General gating strategy to identify live CD14^+^ monocytes. Representative histograms (B) and cumulative data (C) showing the expression of HLA-DR (*n* = 27), CD86 (*n* = 29), CD80 (*n* = 7), PD-L1 (*n* = 7), and PD-L2 (*n* = 5) in healthy donor CD14^+^ monocytes transfected with negative control (miR-CTR) or miR-155 mimic, as assessed by flow cytometry. Data analysed by two-tailed Wilcoxon matched pairs test, ∗*P* < 0.05, ∗∗*P* < 0.01, ∗∗∗*P* < 0.001, ∗∗∗∗*P* < 0.0001.

Synovial CD14^+^ monocytes and HLA^high^ISG15^+^ STM are characterized by antigen-presenting pathways [4, 11, 18, 23], suggesting that miR-155 may drive antigen-presenting properties of synovial macrophages. Thus, we investigated whether miR-155 regulated the expression of MHC II and co-stimulatory/co-inhibitory molecules. Peripheral CD14^+^ monocytes from healthy donors were transfected with miR-155 or negative control mimic (miR-CTR) for 24 h and assessed for the expression of HLA-DR, co-stimulatory molecules CD86 and CD80, and checkpoint molecules PD-L1 and PD-L2. Cells were gated based on viability, FSC^hi^, and CD14^+^ expression (Fig. 2A). Monocytes transfected with miR-155 showed a significant increase in the expression of HLA-DR, CD86, and CD80 compared to negative control mimic. We also observed a significant increase in the expression of PD-L1, but not PD-L2 (Fig. 2B and C). In addition, we observed an increase in the pro-inflammatory cytokines TNFα, IL-6, and IL-8, and in anti-inflammatory IL-10 (Supplementary Figure 6) following miR-155 transfection, thus confirming previous reports [16–18]. It should be noted that whilst a statistically significant increase in IL-10 production was observed following miR-155 transfection, there was considerable variation between donors. Overall, these data support the notion that miR-155 promotes an activated phenotype in CD14^+^ monocytes, typified by the expression of pro-inflammatory cytokines, antigen-presenting and co-stimulatory molecules, as well as negative feedback molecules that limit inflammatory function (IL-10 and PD-L1).

### miR-155-transfected CD14^+^ monocytes promote expression of IFNγ and IL-10 in autologous CD4^+^ T cells

**Fig. 3.**
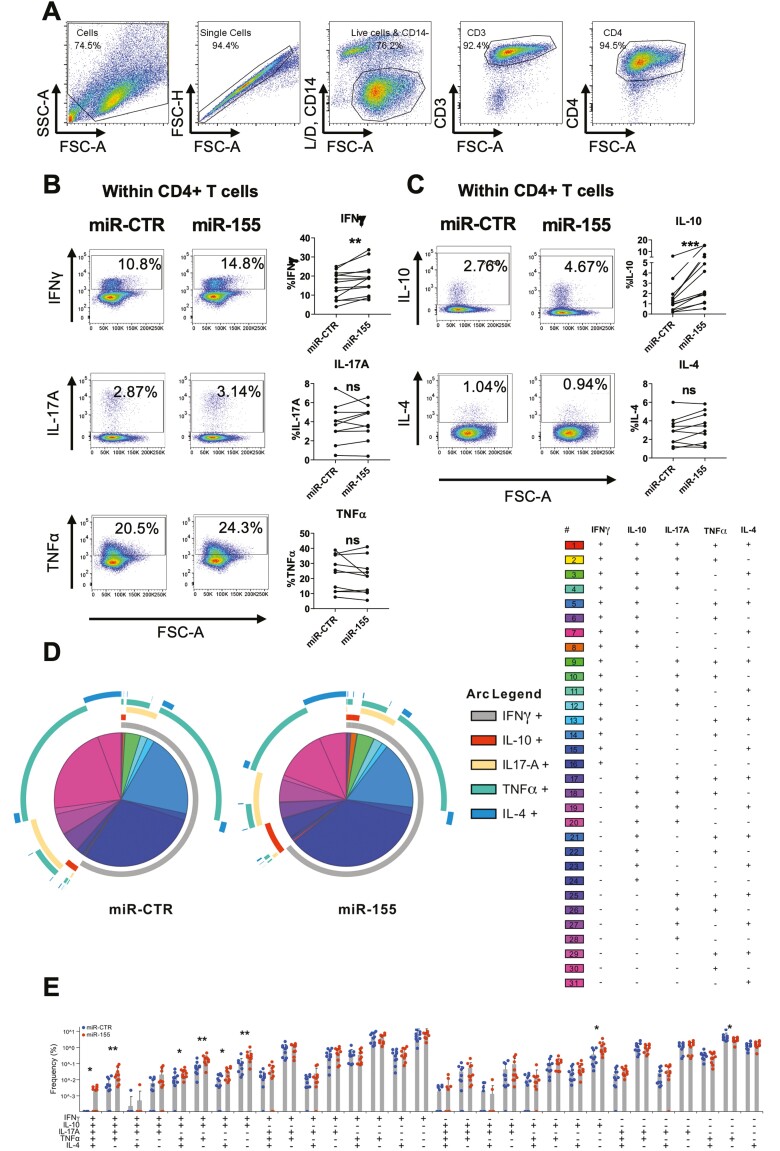
Effect of miR-155-transfected CD14^+^ monocytes on cytokine expression by autologous CD4^+^ T cells. miR-155 and negative control mimic (miR-CTR)-transfected CD14^+^ monocytes were co-cultured with autologous CD4^+^ T cells. After 3 days, cells were stimulated for 3 h with PMA/ionomycin in the presence of GolgiStop. Live CD3^+^ CD4^+^ T cells were gated (A) and the frequency of pro-inflammatory (B) or anti-inflammatory (C) cytokine-producing cells was assessed by flow cytometry (*n* = 10). SPICE analysis of cytokine-producing cells from B to C (D) and cumulative plot showing the frequencies for all combinations of cytokine-producing CD4^+^ T-cell populations (E). Data analysed by Wilcoxon test with two-tailed test, ∗*P* < 0.05, ∗∗*P* < 0.01, ∗∗∗*P* < 0.001.

The observation that miR-155 increased the expression of both co-stimulatory and immune checkpoint molecules in CD14^+^ monocytes, prompted us to assess the effect of miR-155-transfected CD14^+^ monocytes on CD4^+^ T-cell phenotype and function. We first transfected CD14^+^ monocytes with a Dy547-labelled miR-155 mimic for 4 h to verify that this period was sufficient for transfection to occur (Supplementary Figure 7A–C). To ensure that there was no cross-transfection of CD4^+^ T cells with miR-155 mimic, we transfected CD14^+^ monocytes with a Dy547-labelled miR-155 mimic for 4 h, washed the cells and co-cultured them for 24 h with autologous CD4^+^ T cells in the presence of anti-CD3 mAb. After 24 h we confirmed that there was no Dy547-miR-155 mimic in the CD4^+^ T-cell population (Supplementary Figure 7D and E). We then co-cultured CD4^+^ T cells with transfected monocytes for 72 h in the presence of soluble anti-CD3 mAb (100 ng/ml), followed by stimulation with PMA, ionomycin, and Golgistop. CD4^+^ T cells (gating strategy shown in Fig. 3A) cultured in the presence of miR-155-transfected CD14^+^ monocytes showed a significant increase in the frequencies of IFNγ-expressing cells and of IL-10-expressing cells compared to those cultured with miR-CTR-transfected monocytes. There was no significant difference in the frequencies of CD4^+^ T cells expressing IL-17A, TNFα, or IL-4 (Fig. 3B and C). To investigate polyfunctionality of these cells, we used the visualization software SPICE [22]. This revealed that the IFNγ- and IL-10-expressing T cells in the miR-155 condition were mostly polyfunctional CD4^+^ T cells, i.e. co-expressing 2 to 5 cytokines (Fig. 3D and E), although it should be noted that many of these frequencies were low. We also observed a significant increase in the frequency of single IL-10-producing CD4^+^ T cells and a contraction in single TNFα-producing cells (Fig. 3D and E).

**Fig. 4.**
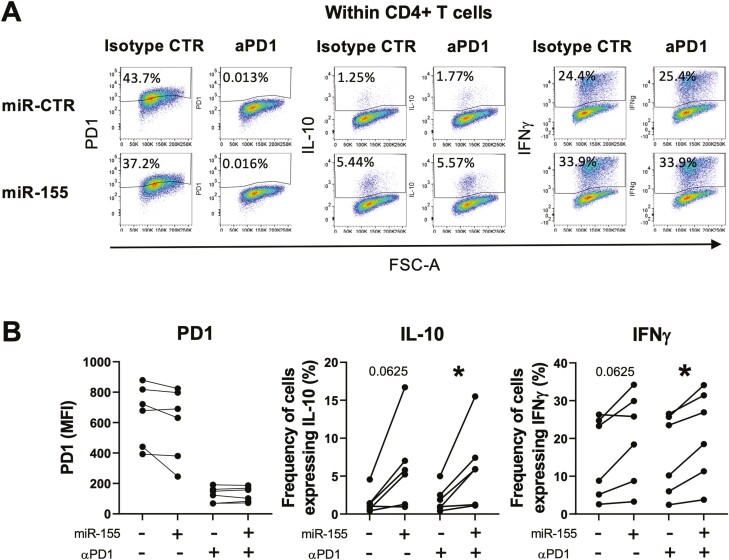
Effect of PD1 blockade on the cytokine profile of CD4^+^ T cells during co-culture with miR-155-transfected CD14^+^ monocytes. miR-155 and negative control mimic (miR-CTR)-transfected CD14^+^ monocytes were co-cultured with autologous CD4^+^ T cells in the presence of the aPD1 mAb pembrolizumab (aPD1) or isotype control mAb (control). After 3 days, cells were stimulated for 3 h with PMA/ionomycin in the presence of GolgiStop and expression of PD1, IL-10, and IFNγ was assessed by flow cytometry (*n* = 6). Representative (A) and cumulative (B) data are shown. Data analysed by Wilcoxon test with two-tailed test, ∗*P* < 0.05.

Since we found that miR-155 increased the expression of PD-L1 in CD14^+^ monocytes (Fig. 2), we investigated whether PD-L1-expressing monocytes may contribute to the increase in IL-10 or IFNγ expression in CD4^+^ T cells by using the PD1-blocking antibody pembrolizumab. CD14^+^ monocytes were transfected and co-cultured with CD4^+^ T cells as described, in the presence of either blocking anti-PD1 or isotype control mAb (1 µg/ml). When cultured in the presence of anti-PD1-blocking antibody, no PD1 expression on CD4^+^ T cells was observed compared to CD4^+^ T cells cultured with the isotype control antibody (Fig. 4A and B), which likely is due to blocking or masking of the epitope by pembrolizumab. As before, an increase in IL-10- and IFNγ-expressing CD4^+^ T cells was observed after co-culture with miR-155-transfected CD14^+^ monocytes, and this was still observed in the presence of the PD1-blocking antibody pembrolizumab (Fig. 4A and B). These data indicate that the activation of polyfunctional CD4^+^ T cells by miR-155-overexpressing CD14^+^ monocytes occurs in a PD-L1-independent manner.

## Discussion

Our data demonstrate that miR-155 overexpression of healthy control blood monocytes confers a gene signature similar to that of RA synovial macrophages. We report that the transcriptional profile of miR-155-transfected monocytes shows significant similarities to the gene expression profile of RA synovial fluid-derived CD14^+^ cells. Further analysis revealed that the gene signature of the HLA^high^ISG15^+^ ST-derived macrophage subset, which is predominantly present in treatment-resistant, active RA [11], is enriched within miR-155-overexpressing monocytes, and that this subset expresses high levels of miR-155. Together with our data that synovial CD14^+^ cells have increased expression of miR-155, and that inflammatory cytokines or cell-free synovial fluid from the RA joint can increase miR-155 expression in monocytes, our study reaffirms the association of miR-155 and joint inflammation in RA. It also reveals that monocytes with high expression of miR-155 can modulate the adaptive immune response by stimulating polyfunctional CD4^+^ T cells.

Monocytes overexpressing miR-155 share phenotypic similarities with HLA^high^ISG15^+^ synovial macrophages (Fig. 1D–F), as miR-155 overexpression in healthy control monocytes leads to increased expression of HLA-DR, as well as of the co-stimulatory molecules CD86 and CD80, and the co-inhibitory molecule PD-L1 (Fig. 2). MiR-155 expression has been shown to be increased during DC maturation supporting the role for miR-155 in antigen presentation [29]. In mice, it was reported that overexpression of miR-155 in the macrophage cell line RAW264.7, treated with LPS or ox-LDL, increased the expression of MHCII and CD86 [30]. Conversely, knockdown of miR-155 in murine Kupffer cells decreased expression of MHCII, CD86, and CD40, and lowered production of TNFα, IL-6, and IL-1β [31, 32]. The increase in HLA-DR and co-stimulatory marker expression suggests that miR-155-transfected monocytes may potentiate T-cell activation. In support of this, we observed an increase in IFNγ^+^ and in IL-10^+^ T cells when CD4^+^ T cells were co-cultured with miR-155-transfected CD14^+^ monocytes (Fig. 3). The increase in IFNγ-producing CD4^+^ T cells supports a role for miR-155 in driving synovial pathology as the inflamed RA and PsA joints harbour a large proportion of IFNγ-producing CD4^+^ T cells [33, 34]. It has also been shown that synovial monocytes from the inflamed RA joint, which express increased levels of miR-155, can polarize CD4^+^ T cells towards a Th1/Th17 phenotype [2, 4]. We previously showed increased production of the Th1-polarizing cytokine IL-12 by miR-155-transfected CD14^+^ monocytes [18], which together with the increased expression of HLA-DR, CD80, and CD86 may contribute to the observed Th1 polarization. These Th1 cells in turn could increase the pro-inflammatory activity of synovial monocytes and macrophages via their production of IFNγ.

In addition to these pro-inflammatory effects, our study provides evidence that miR-155 overexpression simultaneously promotes an immunoregulatory response. This may be caused either by direct targeting of pro-inflammatory genes by miR-155 or indirectly through downstream modulation of immunoregulatory pathways. We show that miR-155-transfected monocytes upregulate IL-10 production and PD-L1 expression, and subsequently promote an increase in IL-10-expressing CD4^+^ T cells upon co-culture (Figs 2 and 3). The co-expression of IL-10 in otherwise pro-inflammatory cytokine-producing CD4^+^ effector T cells has been reported to occur under a variety of conditions [35–38]. A potential reason for the increase in IL-10 could be to limit tissue damage by inhibiting the release of TNFα, IL-1β, IL-6, and IL-8 [39, 40], reducing expression of MHCII and co-stimulatory markers [40, 41] and inhibiting the production of the Th1-polarizing cytokine IL-12 by monocytes and macrophages [42].

Other studies have also investigated the relation between IL-10 and miR-155 in monocytes. In an atherosclerosis setting, peritoneal macrophages from miR-155-deficient mice were more pro-inflammatory and produced less IL-10 following LPS stimulation [43]. A different study reported that LPS-stimulated monocytes exposed to lower temperatures produced less IL-10 and suppressed the expression of SHIP-1 and SOCS1 in a miR-155-dependent manner [44]. Furthermore, it was shown that Kruppel-Like Factor 2 (KLF2) indirectly suppressed miR-155 expression thereby reducing production of pro-inflammatory mediators and increasing the production of IL-10 and SOCS1 in macrophages following stimulation with ox-LDL [45]. Thus, whilst the relation between miR-155 and IL-10 needs further exploration, our collective data support the notion that the expression of miR-155 in synovial monocytes or macrophages contributes to a pro-inflammatory environment in RA. The polarization of IFNγ^+^ CD4^+^ T cells by miR-155-expressing myeloid cells may potentiate inflammation through a positive feedforward loop in which the Th1 cells further activate the synovial myeloid cells. Whilst this may trigger an autoregulatory IL-10-mediated response to reduce collateral tissue damage, the presence of hyperactive macrophages and CD4^+^ T cells is likely to contribute to the ongoing joint inflammation as observed in RA.

## Supplementary Material

uxab016_suppl_Supplementary_Figures_S1-S7_and_Table_S1

## Data Availability

RNA-seq data of miR-155- vs. negative control mimic-transfected monocytes can be accessed at GEO upon publication [GSE166605].

## Abbreviations

miR: microRNA
PBM: peripheral blood monocytes
PBMC: peripheral blood mononuclear cells
PsA: psoriatic arthritis
RA: rheumatoid arthritis
SFM: synovial fluid monocytes
SFMC: synovial fluid mononuclear cells
ST: synovial tissue
STM: synovial tissue macrophage
