# Supplementary material for: miR-155-overexpressing monocytes resemble HLAhighISG15+ synovial tissue macrophages from patients with rheumatoid arthritis and induce polyfunctional CD4+ T-cell activation
Source: Clin Exp Immunol. 2021 Nov 30;207(2):188–98. doi: 10.1093/cei/uxab016 (PMC8982969; doi:10.1093/cei/uxab016)
Supplement: uxab016_suppl_Supplementary_Figures_S1-S7_and_Table_S1 [file uxab016_suppl_supplementary_figures_s1-s7_and_table_s1.pdf]

	RA	PsA
n	14	9
Age (years)*	58 ± 12	37 ± 11
Female, n (%)	8 (57%)	3 (33%)
CRP (mg/L)*	27 ± 28	26 ± 21
ESR (mm/hr)*	38 ± 32	36 ± 37
DAS28*	4.8 ± 0.8	4.1 ± 1.6
no treatment, n (%)	4 (28.6%)	5 (55.6%)
NSAID[1], n (%)	1 (7.1%)	2 (22.2%)
DMARD[2], n (%)	7 (50%)	3 (33.3%)
Biologic[3], n (%)	6 (42.9%)	1 (11.1%)

Supplementary Table 1. Demographic and clinical information for patients included in this study.
* values denote mean ± SD**;** [1] Includes Naproxen and Etoricoxib; [2] Includes methotrexate, hydroxychloroquine and sulfasalazine; [3] Includes adalimumab, baricitinib, certolizumab, abatacept and tocilizumab; CRP, C-reactive protein; DAS28, Disease Actvitiy Score 28 joints; DMARD, disease-modifying anti-rheumatic drug; ESR, erythrocyte sedimentation rate; NSAID, non-steroidal anti-inflammatory drug.

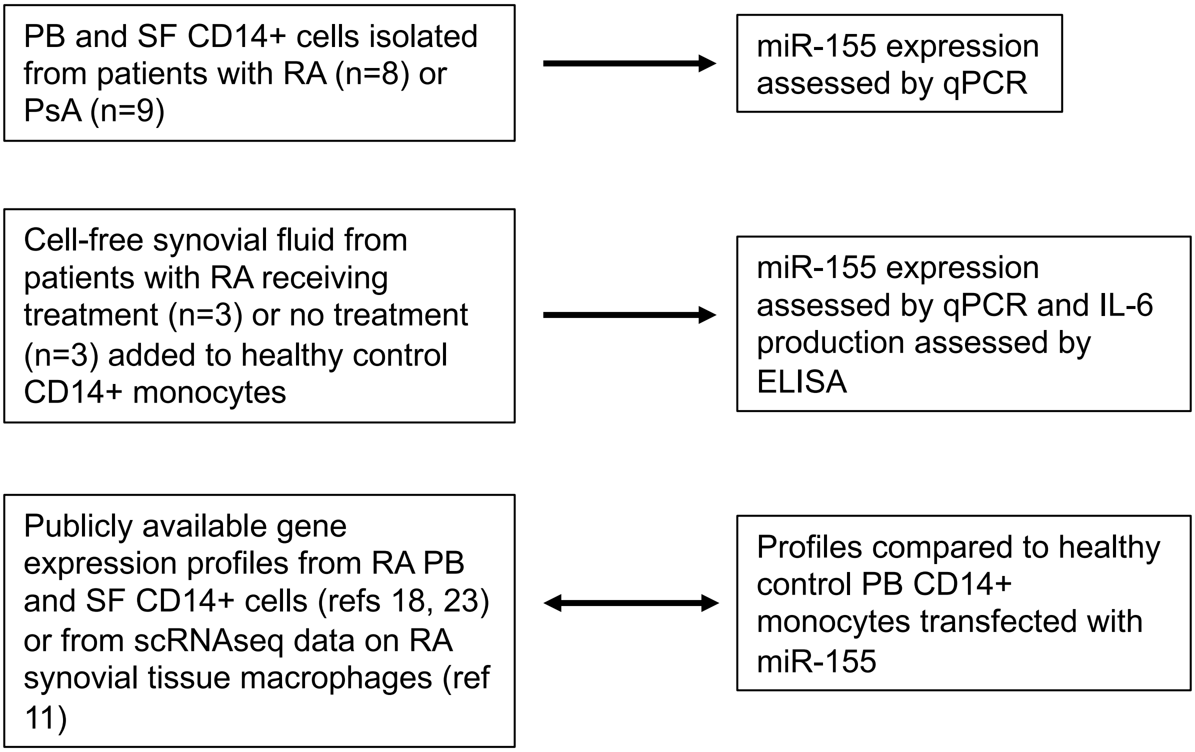

Supplementary Figure 1. Flow chart for use of patient samples.

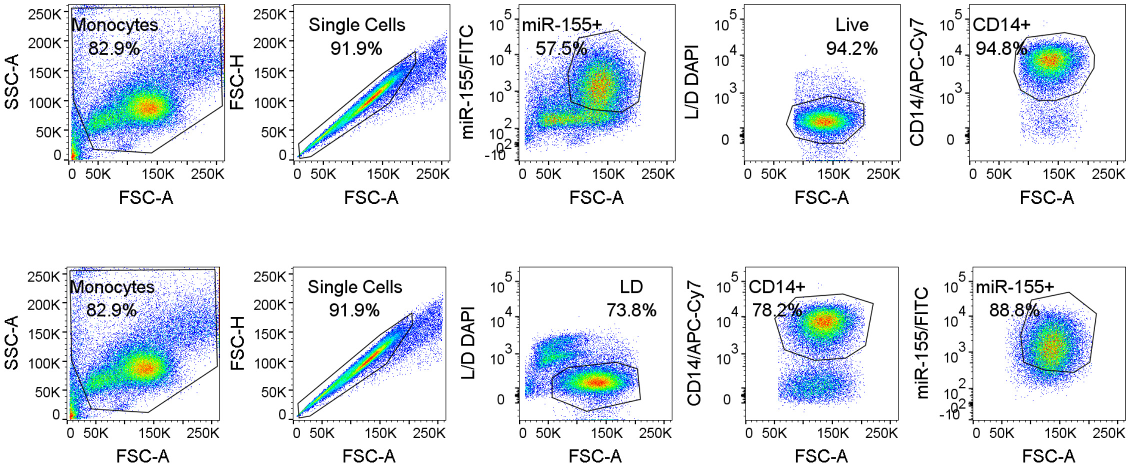

Supplementary Figure 2. Live CD14^{hi} FSC^{hi} monocytes are enriched for miR-155 transfected cells. Gating strategy demonstrating that fluorescently tagged miR-155 transfected monocytes are viable (94.2%, as shown by live/dead dye) and CD14^{high} (94.8%) (upper panel), and vice versa, that live CD14^{high} FSC^{hi} monocytes contain a high proportion of transfected cells (88.8%) (lower panel).

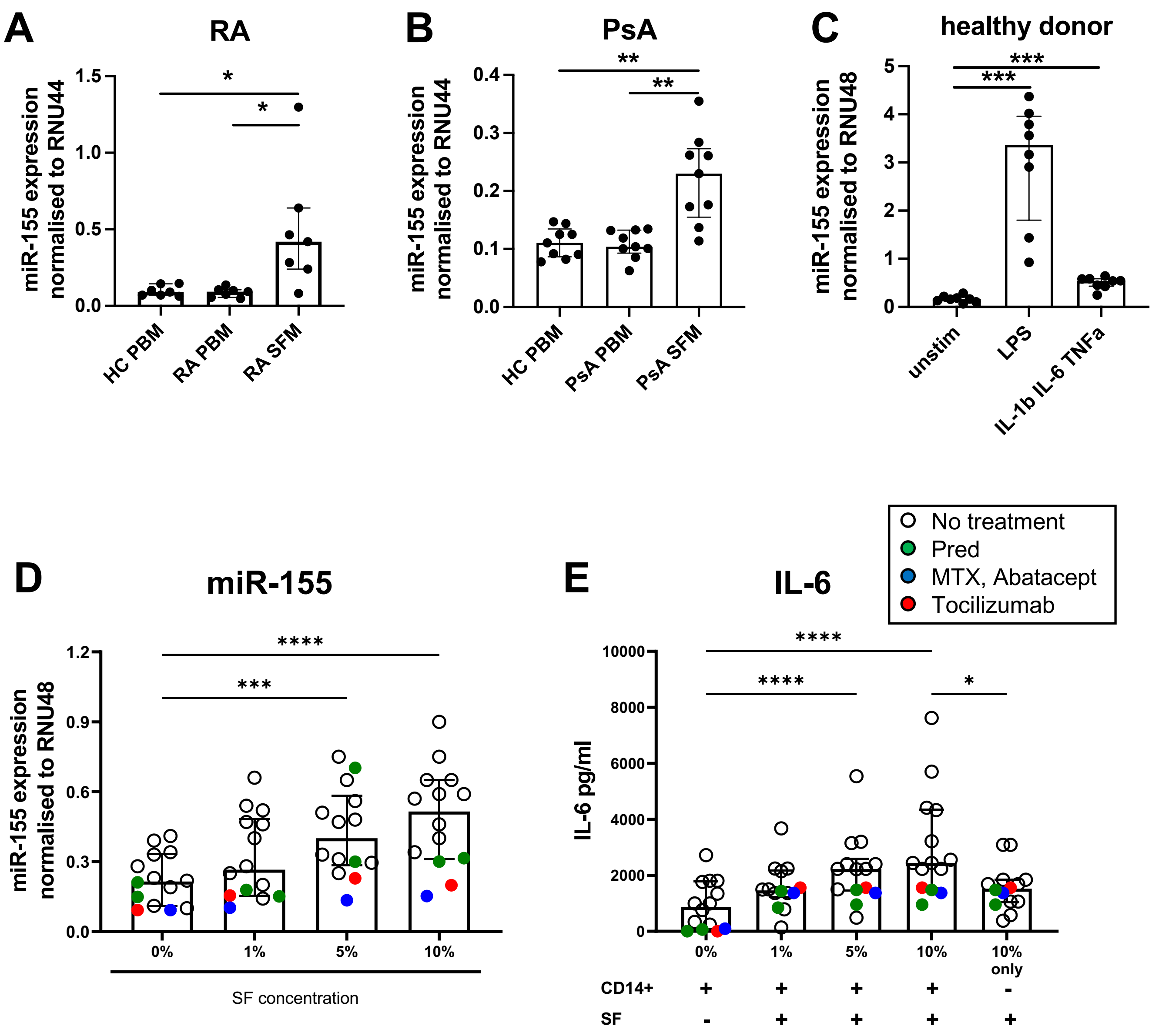

Supplementary Figure 3. Expression of miR-155 in RA and PsA synovial CD14+ cells and in response to pro-inflammatory stimuli. (A, B) Expression of mature miR-155 was measured in CD14+ peripheral blood monocytes (PBM) of healthy controls (A, n=7; B, n=9) or in paired PBM and synovial fluid monocytes (SFM) from patients with RA (A, n=7) or PsA (B, n=9) by qPCR. Expression was normalised to RNU44. Data analysed by Kruskal-Wallis test with Dunn's multiple comparison. (C) Healthy donor CD14+ monocytes were cultured for 24 hours in the absence or presence of LPS (50 ng/ml) or a combination of IL-1β, IL-6 and TNFα (10 ng/ml each) (n=8), Results were analysed by ANOVA. (D, E) Healthy donor CD14+ monocytes were cultured for 24 hours in the absence or presence of cell-free SF from patients with RA (n=2 different HC treated with n=3 different RA SF, and n=2 different HC treated with n=2 different RA SF). Following culture, mature miR-155 expression was measured by qPCR relative to RNU48 (D) or supernatants were collected and production of IL-6 was measured by ELISA (E). Open symbols indicate SF from drug-free patients. Green symbols indicate SF from prednisolone, blue symbols from MTX and abatacept, and red symbols from tocilizumab treated patients. Results were analysed by Friedman test with Dunn's multiple comparison. * $p<0.05$, **** $p<0.0001$.

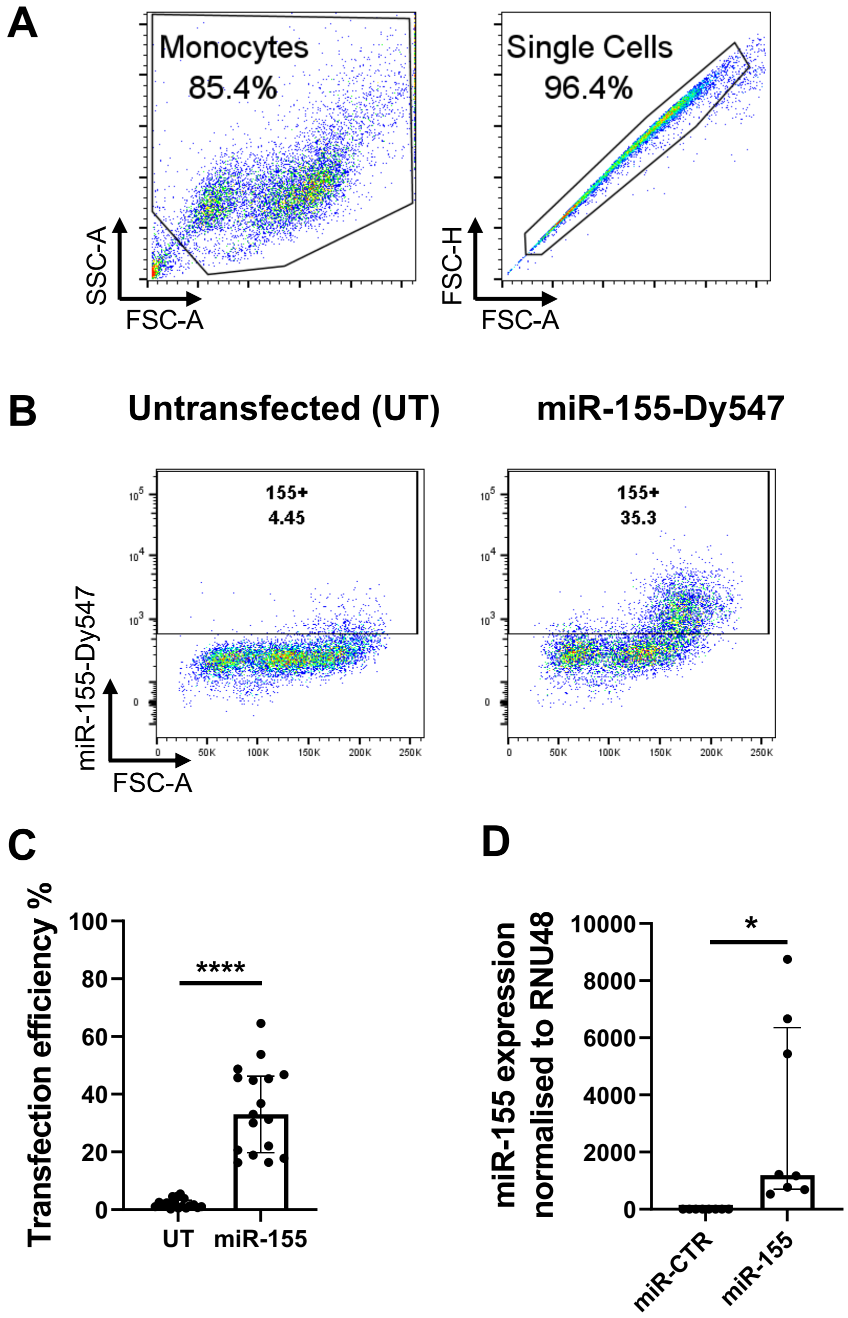

Supplementary Figure 4. miR-155 transfection efficiency in CD14+ monocytes. (A) Representative gating strategy of CD14+ monocytes from PB of healthy controls 24 hours after transfection. (B, C) Monocytes were left untransfected (UT) or transfected with a Dy547-labelled miR-155 mimic to assess transfection efficiency at 24 hours (read out on FITC channel). Representative (B) and cumulative data (C, n=17) showing the transfection efficiency in miR-155-Dy547 transfected CD14+ monocytes. (D) The expression of mature miR-155 was assessed by qPCR in negative control (miR-CTR) or miR-155 mimic transfected CD14+ monocytes (n=9) and normalised to RNU48. Data analysed by two-tailed paired t-test, * $p<0.05$ **** $p<0.0001$.

A

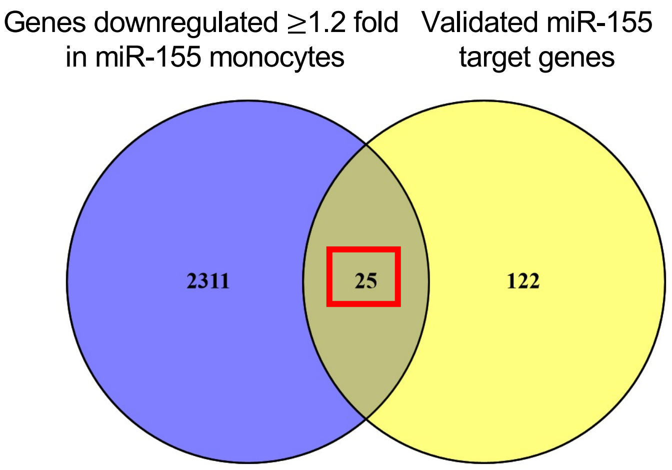

B

Gene	fold change	adjusted p value
ANAPC16	-1.3645923	0.00143612
APAF1	-1.3457779	0.00215994
CARD11	-2.4647529	0.03481587
CARHSP1	-1.4952975	0.00073849
CLUAP1	-1.6656955	0.04613225
EXOSC2	-1.5814718	0.0014315
FAM135A	-1.610749	3.86E-05
FOS	-1.534636	0.00017904
GABARAPL1	-1.2371336	0.03240061
HDAC4	-1.5048053	0.00397724
JADE1	-1.3346526	0.03851315
MBNL3	-1.958204	3.33E-05
MRPS27	-2.0165555	1.22E-07
MYO1D	-1.9590666	0.00248378
NARS	-1.386016	0.00493419
PALD1	-8.2433213	6.08E-06
PAXBP1	-1.2757809	0.04453179
PHC2	-1.2959605	0.0054353
POLE3	-1.3575863	0.0048276
S1PR1	-3.4119519	7.63E-05
SKI	-2.4979081	1.25E-07
TOMM20	-1.4750349	0.00102788
TP53INP1	-1.5405097	0.00016474
ZNF248	-2.0324912	2.75E-05
ZNF652	-1.6093783	5.99E-05

C

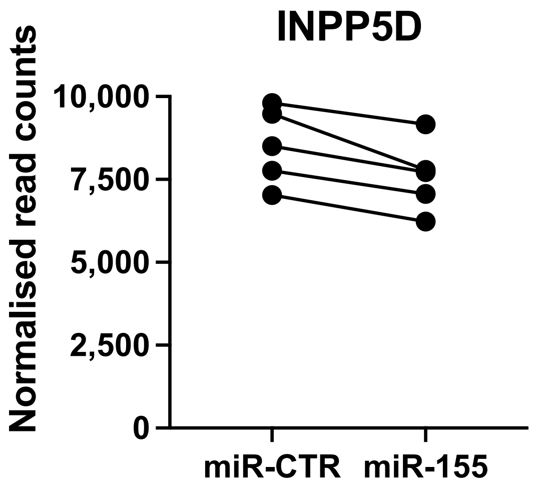

Supplementary Figure 5. miR-155 transfected CD14+ monocytes show downregulation of miR-155 target genes. CD14+ monocytes were transfected for 24 hours with miR-155 or negative control (miR-CTR) mimic, after which RNA was isolated for RNAseq. (A) Venn Diagram of number of genes downregulated at least 1.2 fold in miR-155 transfected CD14+ monocytes and validated target genes of miR-155. (B) The list of genes in the cross-section is shown. Fold change is as compared to negative control transfected CD14+ monocytes. (C) Normalised expression in five donors of the miR-155 target gene *INPP5D* (which encodes SHIP-1). Significance was calculated using Deseq2 on a FDR of 0.05.

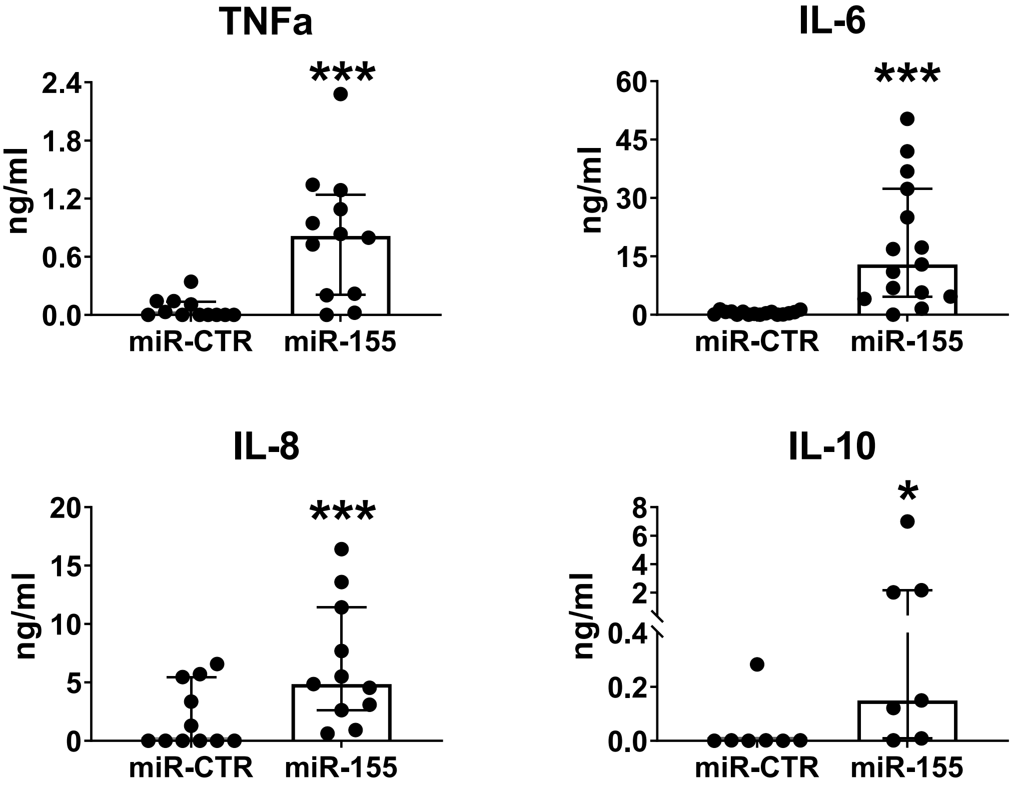

Supplementary Figure 6. Effect of miR-155 overexpression on CD14+ monocyte cytokine production. Healthy donor CD14+ monocytes were transfected with negative control (miR-CTR) or miR-155 mimic and cultured for 24 hours. Production of TNFα (n=12), IL-6 (n=17), IL-8 (n=11) and IL-10 (n=7) was measured by ELISA. Data analysed by two-tailed Wilcoxon matched pairs test.

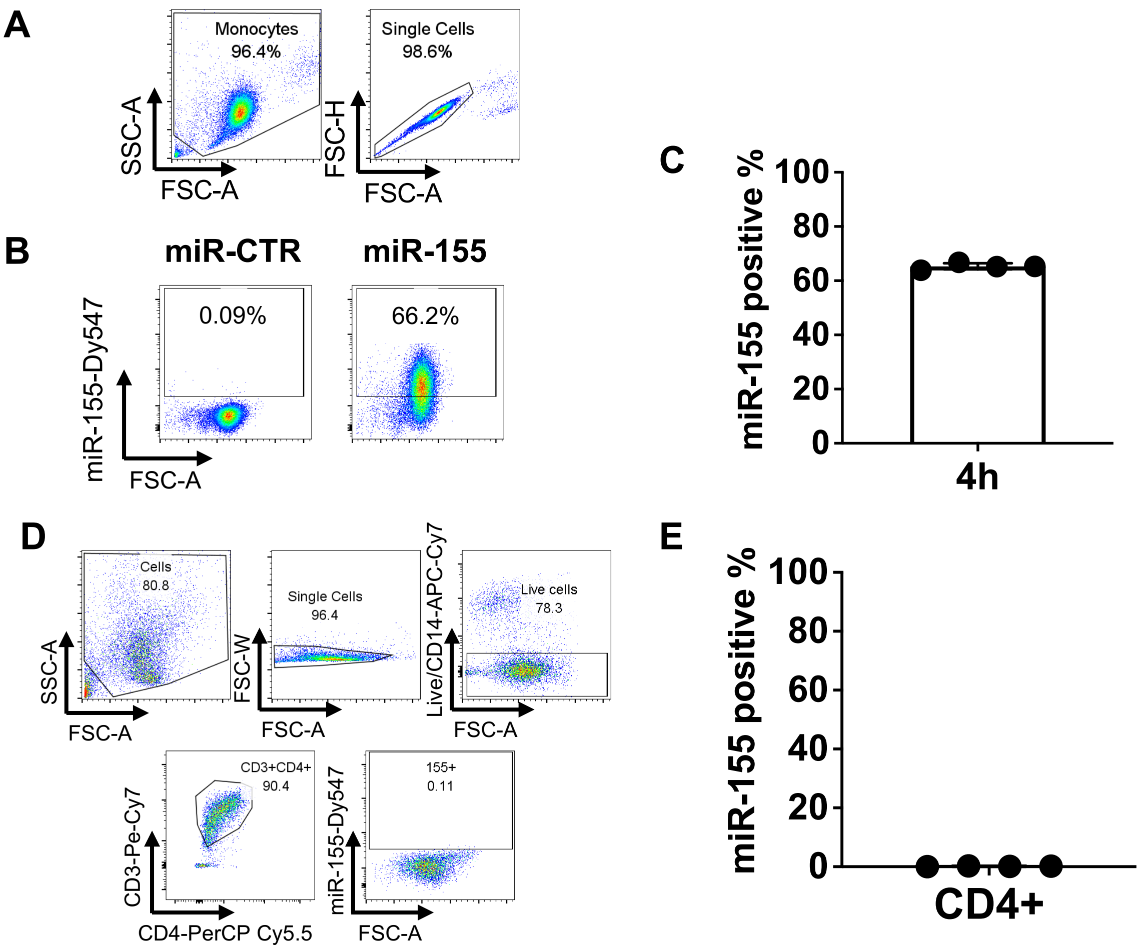

Supplementary Figure 7. miR-155 transfection of CD14+ monocytes does not lead to subsequent cross-transfection of autologous CD4+ T cells

Healthy donor MACS-isolated CD14+ monocytes were transfected with negative control (miR-CTR) or miR-155-Dy547 for 4h. (A) General monocyte gating strategy, with representative (B) and cumulative (C, n=4) data showing the transfection efficiency at 4h in CD14+ monocytes transfected with negative control (miR-CTR) or miR-155-Dy547 (read out on the FITC channel). (D, E) Following 4h transfection, CD14+ monocytes were washed to remove excess transfection mix and co-cultured with autologous CD4+ T cells for 24h in the presence of αCD3 mAb. After 24h, CD4+ T cells were stained with CD3-PE/Cy7, CD4-PerCP/Cy5.5 and Live/CD14-APC/Cy7. Following staining, live CD3+CD4+ T cells were gated and assessed for miR-155 expression. Representative (D) and cumulative data (E, n=4) showing the absence of miR-155 expression in CD4+ T cells at 24h. Data are shown as median with interquartile range.
